# Evaluation of Video-Assisted Thoracoscopic Surgery for Pulmonary Metastases: A Meta-Analysis

**DOI:** 10.1371/journal.pone.0085329

**Published:** 2014-01-09

**Authors:** Siyuan Dong, Lin Zhang, Wenya Li, Jiang Du, Xiangli Liu, Xitao Chen

**Affiliations:** Department of Thoracic Surgery, First Hospital of China Medical University, Shenyang, Liaoning Province, People's Republic of China; University of Kansas, United States of America

## Abstract

**Background:**

To evaluate the evidence comparing video-assisted thoracic surgery (VATS) and open thoracotomy in the treatment of metastatic lung cancer using meta-analytical techniques.

**Methods:**

A literature search was undertaken until July 2013 to identify the comparative studies evaluating disease-free survival rates and survival rates. The pooled odds ratios (OR) and the 95% confidence intervals (95% CI) were calculated with the fixed or random effect models.

**Results:**

Six retrospective studies were included in our meta-analysis. These studies included a total of 546 patients: 235 patients were treated with VATS, and 311 patients were treated with open thoracotomy. The VATS and the thoracotomy did not demonstrate a significant difference in the 1-,3-,5-year survival rates and the 1-year disease-free survival rate. There were significant statistical differences between the 3-year disease free survival rate (p = 0.04), which favored open thoracotomy.

**Conclusions:**

The VATS approach is a safe and feasible treatment in terms of the survival rate for metastatic lung cancer compared with the thoracotomy. The 3-year disease-free survival rate in the VATS group is inferior to that of open thoracotomy. The VATS approach could not completely replace open thoracotomy.

## Introduction

Metastasectomy is considered a beneficial treatment for a patient with metastatic lung cancer whose primary tumor has been well controlled[1].After surgery, 5-year survival rates of 30% to 50% could be achieved depending on the underlying primary cancer[2]–[4].In practice, the surgical approaches to pulmonary metastases are variable. Video-assisted thoracoscopic surgery (VATS) is an emerging technique; many procedures that had previously required a thoracotomy have been performed with the minimally invasive VATS. VATS has been used for the treatment of pulmonary metastases.

The routine use of VATS for the treatment of respectable metastatic lung cancer remains controversial. Critics of the VATS approach have argued that it might not be an equivalent oncological operation[5], [6]. A prospective study by Cerfolio[7]found that 22% of the nodules that could be detected by thoracotomy were missing by VATS.Whether the VATS approach can provide a satisfactory outcome is unknown.

An evidenced-based investigation of the VATS approach is needed, we undertook this meta-analysis to achieve a more objective assessment of the published studies and to provide a more accurate comparison between VATS and thoracotomy for metastatic lung cancer.

## Methods

### Search Strategy

Electronic searches were of the MEDLINE,Cochrane Controlled Trial Register (CENTRAL), Ovid MEDILINE, PubMed and Embase databases were performed until July 2013.The following MeSH search headings were used: “metastatic lung cancer”, “pulmonary metastases” “video-assisted thoracic surgery”, “thoracotomy” and “comparative study”.We searched the reference lists of relevant studies, reviews, editorials, letters,and meeting abstracts. We used the Science Citation Index to cross-reference for further studies that met our criteria.

### Study Selection

The studies included in this meta-analysis were based on our predetermined criteria as follows: (1) clinical trials that include the full text of the paper published in peer-reviewed English journals or reports of presentations at major thoracic surgery meetings; (2) comparison of the efficacy of VATS to that of thoracotomy in patients with metastatic lung cancer; and (3) similarity in the patients' baseline characteristics.

### Data extraction and quality assessment

Two independent reviewers (Siyuan and Wenya) assessed the quality and the risk of bias of the included trials as follows: (1) the studies that did not include a comparative group with surgery as a form of intervention were excluded; (2) the trials focusing on patients undergoing surgery for primary lung cancer were excluded; (3) the studies on robotic video-assisted thoracic surgery were excluded; (4) if there was an overlap between authors, centers or patient cohorts evaluated in the published literature, only the most recent report was included; (5) studies published more than 20 years ago were excluded because of the significant technological changes that has occurred. The articles were evaluated with the Downs and Black quality assessment method[8]. Discrepancies between the two investigators were resolved by discussion and consensus with a senior investigator. The final results were reviewed by two senior investigators (Lin and Jiang).The disease-free survival was defined as the date of the initial metastasectomy until the date of a recurrence.

### Statistical and sensitivity analyses

The meta-analysis was performed using the RevMan 5.1.0. software package. The odds ratio (OR) or the mean difference with 95% confidence intervals (95% CI) was calculated for the dichotomous outcomes and the continuous outcomes, respectively. A P value<0.05 was considered a significant difference in the value between the two groups. We used the I^2^ statistic to investigate the heterogeneity among the studies.The heterogeneity was explored by X^2^ and I^2^; I^2^<25% and I^2^>50% reflect a small and large inconsistency, respectively. *P*<0.05 was considered significant. If there were a statistical difference in terms of the heterogeneity (P≤0.05), a random-effect model was selected to pool the data. Otherwise, a fixed-effect model was used. Taking into account the presence of different sample sizes of the included studies, a sensitivity analysis was performed to compare the of 1-year survival rate and the 3-year disease free survival rate between VATS and open thoracotomy.

### Publication bias

A funnel plot was used to explore bias. Asymmetry in the funnel plot of trial size against treatment effect was used to assess the risk of bias.

## Results

### Description of the studies

**Figure 1:**
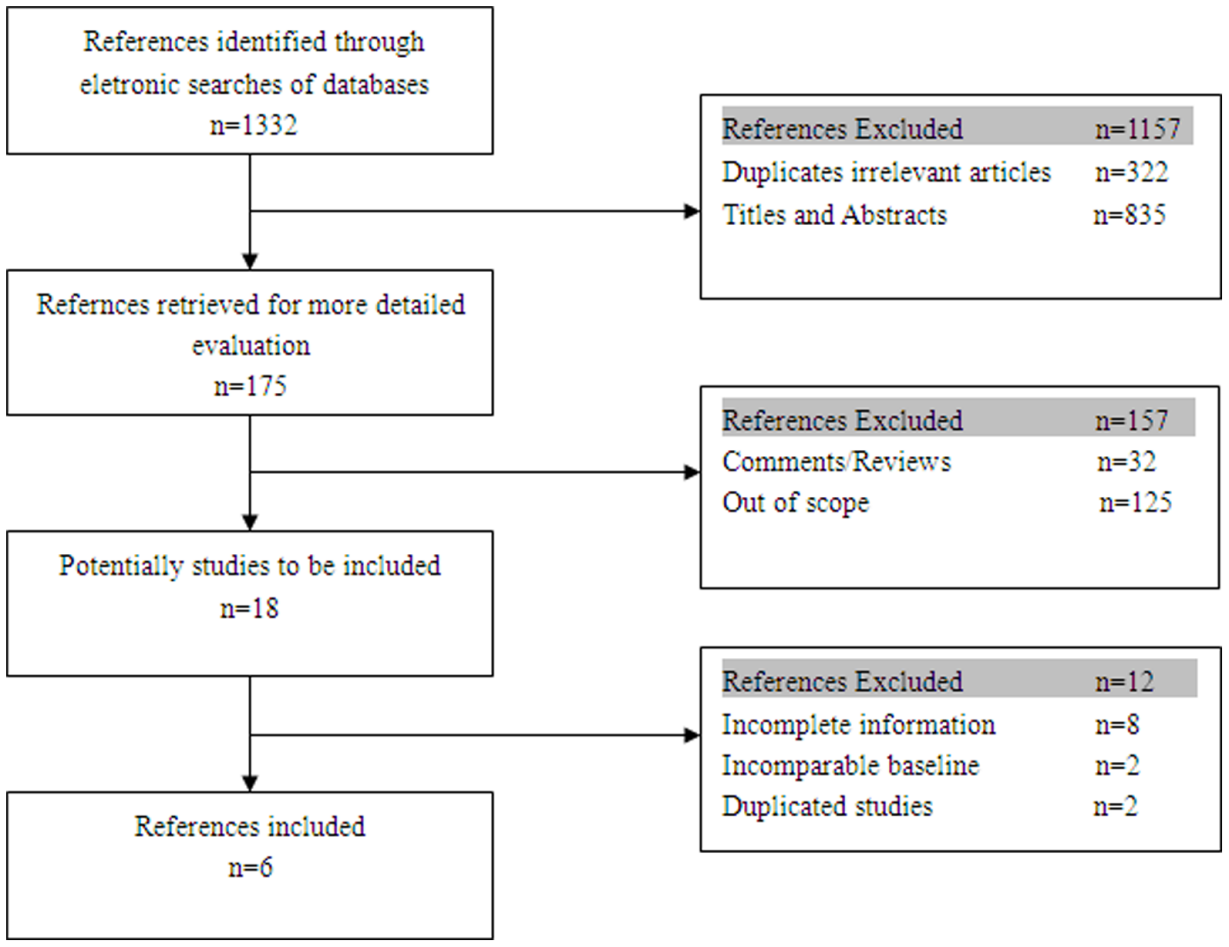
Identification of studies for inclusion.

Six retrospective cohort studies the met our criteria were included in this meta-analysis. A total of 546 patients were included in the six studies;235 patients were allocated to the VATS group, whereas 311 were allocated to the open thoracotomy group to evaluate their survival rate.The search algorithm, results of the search strategies and selection criteria are shown in **Fig 1**. The patient characteristics and evaluation index are shown in **Table 1**.

**Table 1:** 

Study	Design	Country	NO(V/O)	Gender (M/F)	Mean age (years)	Assessment score
Nakajima2001[28]	OC	Japan	45/55	V59/41 O34/21	V55±15 O55±14	13
Mutsaerts2002[29]	OC	Netherlands	8/12	NR	NR	19
Nakas2009[30]	OC	UK	25/27	V16/9 O 19/8	V69 O66	16
Carballo2009[31]	OC	USA	36/135	V18/18 O82/53	V58.5 O49	15
Gossot2009[32]	OC	France	31/29	V21/10 O13/16	V43 O40	18
Chao2012[33]	OC	Taiwan	90/53	V49/41 O35/18	NR	13

V, VATS; O, Open thoracotomy; NR, Not reported; OC, observational cohort.

### Assessment of Recurrence and Survival

**Figure 2:**
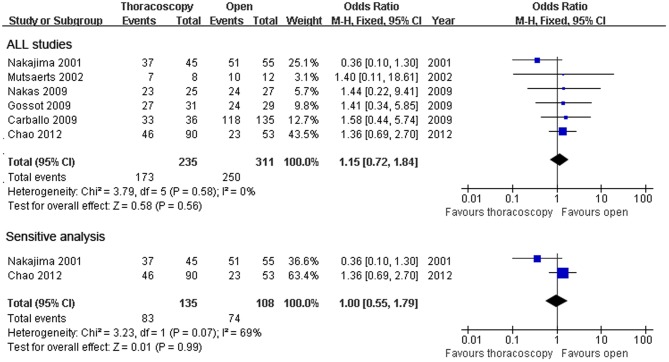
1-year survival rate. Forest plot of the Odds Ratio(OR) of the 1-year survival rate following VATS versus open thoracotomy for metastatic lung cancer.The estimate of the OR of each individual trial corresponds to the middle of the squares and horizontal line gives the 95% CI.On each line,the numbers of events as a fraction of the total number randomized are shown for both treatment groups.For each subgroup,the sum of the statistics, along with the summary OR is represented by the middle of the solid diamonds.A test of heterogeneity between the trials within a subgroup is given below the summary statistics.

**Figure 3:**
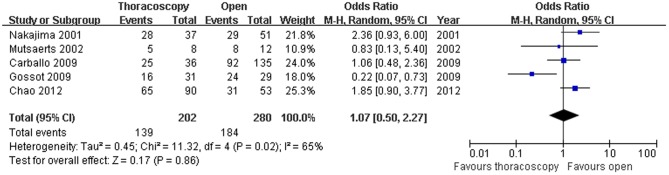
3-year survival rate. Forest plot of the Odds Ratio(OR) of the 3-year survival rate following VATS versus open thoracotomy for metastatic lung cancer.The estimate of the OR of each individual trial corresponds to the middle of the squares and horizontal line gives the 95% CI.On each line,the numbers of events as a fraction of the total number randomized are shown for both treatment groups.For each subgroup,the sum of the statistics, along with the summary OR is represented by the middle of the solid diamonds.A test of heterogeneity between the trials within a subgroup is given below the summary statistics.

**Figure 4:**
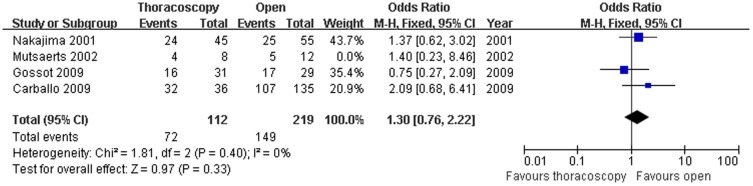
5-year survival rate. Forest plot of the Odds Ratio(OR) of the 5-year survival rate following VATS versus open thoracotomy for metastatic lung cancer.The estimate of the OR of each individual trial corresponds to the middle of the squares and horizontal line gives the 95% CI.On each line,the numbers of events as a fraction of the total number randomized are shown for both treatment groups.For each subgroup,the sum of the statistics, along with the summary OR is represented by the middle of the solid diamonds.A test of heterogeneity between the trials within a subgroup is given below the summary statistics.

Six studies documented the 1-year survival rate,and there was no significant heterogeneity among the six studies (x^2^ = 3.79, P = 0.58,I^2^ = 0%).A fixed effect model was used.The combined result is shown in **Fig 2**(OR = 1.15; 95%CI, 0.72–1.84; p = 0.58). Because of the heterogeneity in sample size, the sensitivity analyses were conducted using larger sample sizes. There was no difference between the two surgical methods with an OR of 1.00(95%CI 0.55–1.79) and with heterogeneity(Χ^2^ = 3.23,P = 0.07, I^2^ = 69%). Five studies reported the 3-year survival rate, and heterogeneity was identified through the five studies (x^2^ = 11.32,P = 0.02,I^2^ = 65%); and a random effect model was adopted (OR = 1.07; 95%CI, 0.50–2.27; p = 0.86) **(Fig 3)**. Three studies compared the 5-year survival rate (OR = 0.96; 95%CI, 0.34–2.71; p = 0.93), with certain heterogeneity(x^2^ = 8.86,P = 0.01,I^2^ = 77%) (**Fig 4**).

**Figure 5:**
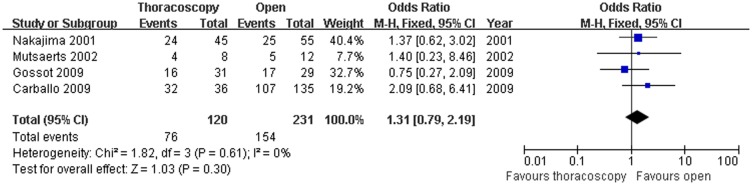
1-year disease-free survival rate. Forest plot of the Odds Ratio(OR) of the 1-year disease free survival rate following VATS versus open thoracotomy for metastatic lung cancer.The estimate of the OR of each individual trial corresponds to the middle of the squares and horizontal line gives the 95% CI.On each line,the numbers of events as a fraction of the total number randomized are shown for both treatment groups.For each subgroup,the sum of the statistics, along with the summary OR is represented by the middle of the solid diamonds.A test of heterogeneity between the trials within a subgroup is given below the summary statistics.

**Figure 6:**
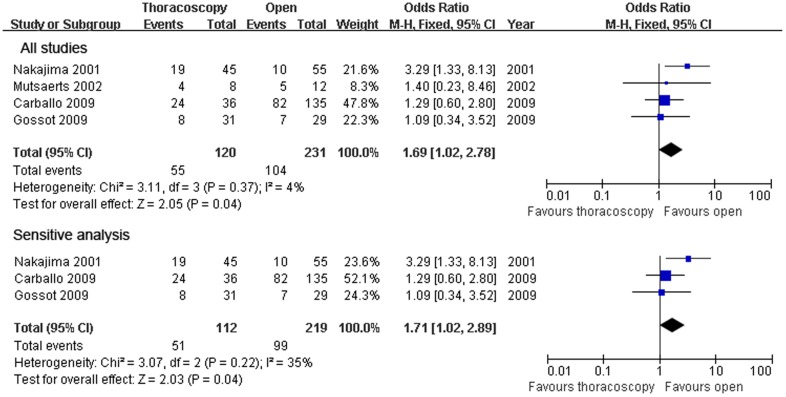
3-year disease-free survival rate. Forest plot of the Odds Ratio(OR) of the 3-year survival rate following VATS versus open thoracotomy for metastatic lung cancer.The estimate of the OR of each individual trial corresponds to the middle of the squares and horizontal line gives the 95% CI.On each line,the numbers of events as a fraction of the total number randomized are shown for both treatment groups.For each subgroup,the sum of the statistics, along with the summary OR is represented by the middle of the solid diamonds.A test of heterogeneity between the trials within a subgroup is given below the summary statistics.

Four studies compared the 1-year disease free survival rate (OR = 1.31; 95% CI, 0.79–2.19; p = 0.30),finding no significant heterogeneity among these studies (x^2^ = 1.82,P = 0.61,I^2^ = 0%) (**Fig 5**), and four studies compared the 3-year disease free survival rate (OR = 0.59; 95% CI,0.38–0.91; p = 0.02), finding no significant heterogeneity (x^2^ = 1.82,P = 0.61,I^2^ = 0%) between the patients who underwent VATS and those who underwent open thoracotomy (**Fig 6**). Because of the heterogeneity in the sample size, sensitivity analyses were conducted using larger sample size studies; however, there was no difference between the two surgical methods with an OR of 1.71 (95% CI,1.02–2.89) and with heterogeneity (Χ^2^ =  3.07,P  = 0.22, I^2^ = 35%). There were significant 3-year disease free survival rate benefits with open thoracotomy. We attempted to evaluate the 5-year disease free survival rate.Only two studies reported these rates,and the published data were not sufficient for the combined analysis.

### Publication bias

**Figure 7:**
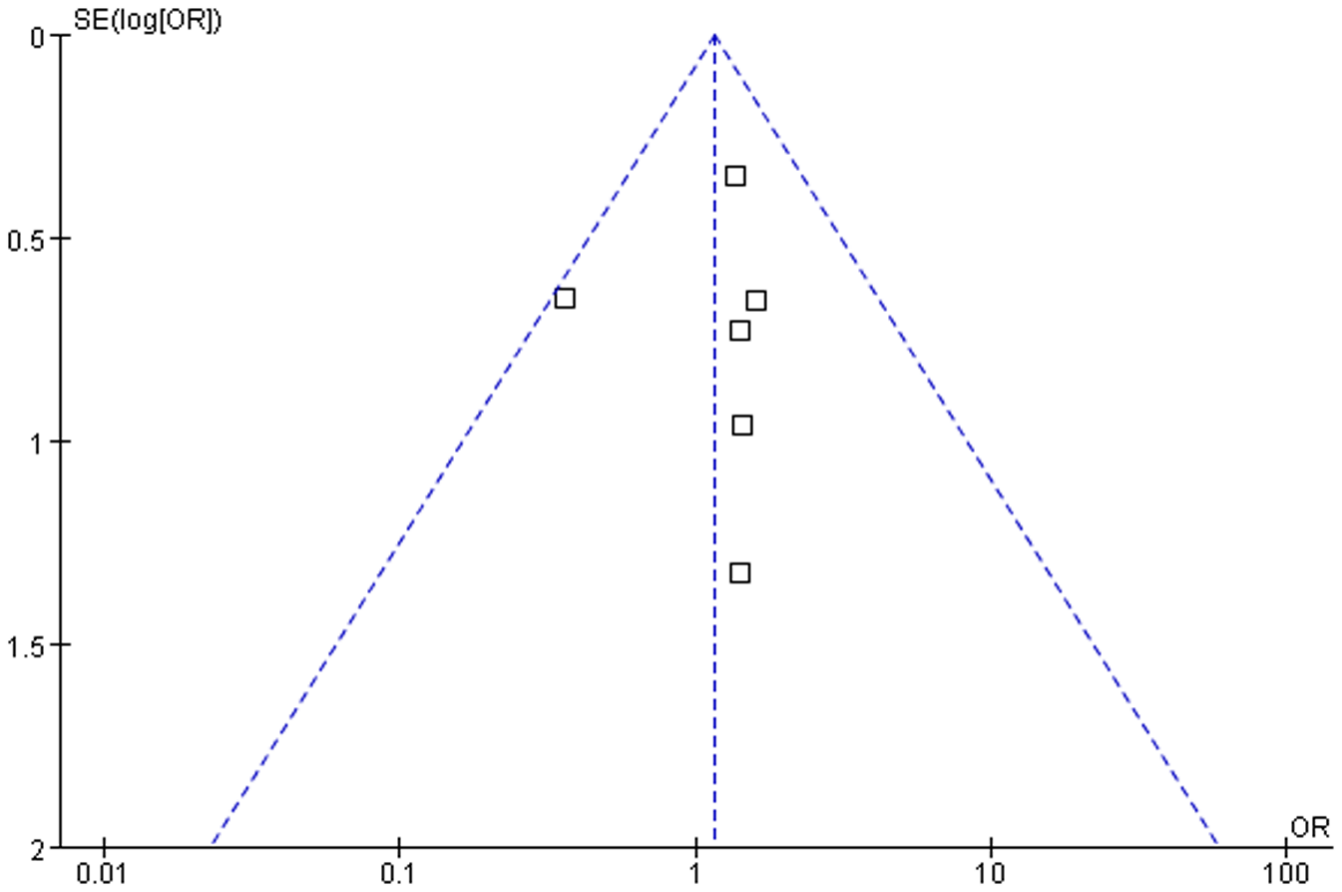
Funnel plot of the outcome of 1-year survival rate.

Publication bias might exist when nonsignificant findings remain unpublished,thus artificially inflating the apparent magnitude of an effect.The funnel plots of the study are shown in **Figure 7**.The funnel plots of the 1-year survival rate following VATS and thoracotomy for the treatment of metastatic lung cancer showed asymmetry, which suggested that there was some publication bias.

## Discussion

Many tumors can metastasize to the lung,and colorectal and breast tumors are the most common primary tumors[9].Pulmonary resection has been shown to be beneficial for patients with resectable and isolated pulmonary metastases[10]. Traditional open thoracotomy and VATS are two principally different surgical methods for pulmonary metastasectomy.The selection of an approach depends more on the theoretical knowledge and personal experience of the surgeon than on the evidence. Over the past two decades, several studies have demonstrated the benefits of VATS that included less postoperative pain, shorter hospital stays, a smaller degree of immunosuppression and enhanced recovery and the ability to tolerate adjuvant therapy[11]–[13]. Whether the long-term advantages are comparable to those of open thoracotomy is not well documented.

The major deficiency of the VATS approach is that nodules might be undetected by VATS that might be detected by manual palpation during thoracotomy; such missing nodules are not imaged on a preoperative CT scan. The VATS approach has long been controversial because VATS does not consistently detect all the metastases, and it is recognized that complete resection remains a major determining factor of survival [14].The detection rate of HRCT for pulmonary metastases is 78–84%[15]–[17].Kayton[18]found that 35% of the pathologically verified metastases were missed by CT. In the International Registry of Lung Metastases study of 5206 patients, the 5-year survival rate was 36% for complete resection compared with 13% incomplete resectoin[19]. It is not certain whether the nodule imaged on a CT scan and resected by VATS is the correct one [14]. Those who disagree with the use of VATS hypothesize that VATS-related recurrence is commonly observed, including port-site recurrence and resection stump recurrence[20]. Johnstone reported 23 cases of port-site chest wall recurrence related to VATS[21]. They hypothesized that the thoracoscopic approach should only be used in patients with a solitary lesion and when resection is requried for diagnostic purposes.

The surgeons who favor the VATS approach advocate that VATS minimizes pain and trauma to the patients and that the VATS group might have an improved tolerance of chemotherapy, which would likely ensure delivery of planned post-resection adjuvant therapy without a reduction in dosage or delay. The standard surgical procedure for pulmonary metastases is wedge resection that usually does not require manipulation of the pulmonary hilum, which is appropriate for the VATS approach.They hypothesiezd that a lesion overlooked by CT but detected by palpation might not result in a survival gain[22], [23], and may be partially compensated for by carefully follow-up.Flores[24] hypothesized that the VATS group might demonstrate a great number of metachronous tumors over time;however, the metachronous lesions in each group was similar.

Our work suggests that thoracoscopic resection of metastatic lung cancer is a safe and curative procedure with 1, 3, and 5-year survival rates comparable to those of thoracotomy. Patients with metastatic lung cancer are likely to relapse in the lung, and after lung metastasectomy by VATS, patients might benefit from a second metastasectomy. We hypothesize that earlier chemotherapy and radiation are essential to maximizing survival. Our study might be subject to pretreatment selection bias, because most of the patients selected for open thoracotomy had multiple lesions and high risk and were not suitable for treatment with VATS.The missing lesions perhaps skewed the data more toward VATS as an equivalent procedure.

We were also interested in the recurrence of cancer,and the disease-free survival rates were evaluated. This study demonstrates a similar 1-year disease-free survival rate;however, the 3-year disease-free survival rate is inferior for three reasons. First, unrelated cancer deaths were included in our analysis of the 1, 3, and 5-year overall survival, which might account for VATS having a comparable overall survival rate but an inferior disease-free survival rate. Second,the patients in the VATS group might have lesions that are missed and there are more likely to relapse in the lung, leading to the inferior 3-year disease-free survival rate.Third, some of our included studies were in the early period of VATS development when the technology was immature, and some of the complications can now be prevented with more experience. Schaeff[25] reported 23 cases of port-site recurrence associated with VATS that occurred before 1998.The number of cases studied was small, and the observation period was limitied.

Spiral computed tomography has a far higher detection rate today than it did 20 years ago;so small lesions can be accurately localized before surgery[26], which ensures the success of VATS. With advances in imaging technology, palpaiton during open thoracotomy is becoming less important.The latest VATS technology has a high-definition resolution and the flexible-tip thoracoscope enables complete inspection of the pleural cavity.These advancements ensure that VATS is an ideal method for patients with a solitary and relatively small peripheral lesions.Tamas[27] hypothesizes that palpation is necessary in a therapeutic metastasectomy as opposed to a diagnostic procedure.Whether patients with multiple lesions should be treated with open thoracotomy or VATS is controversial.

This study is the first meta-analysis of the oncological outcome of thoracoscopic surgery for the treatment of metastatic lung cancer. In our work, we observed that VATS might be a promising treatment for metastatic lung cancer. No randomized trials existing to guide doctors in the field of metastatic lung cancer currently. A prospective randomized study of the different surgical strategies is needed.

### Limitation

No randomized controlled trials existing to comparing VATS with thoracotomy have been conducted. Heterogeneity was observed between the sample size and the years covered. Most studies are limited to small observational studies and single-institution case series. For these reasons,there are only a total of 546 patients were included in the two groups, for a study period spans more than a decade. Two of the studies comprise almost 65% of the patients, and one study has only 20 patients; there are potential sources of bias in our work.Additional randomized controlled trials in the studies we accessed would have increased the strength of our results.There is a bias for the English language.

### Conclusion

In our meta-analysis we found that for patients with metastatic lung cancer, comparing VATS with thoracotomy showed almost equivalent survival rates. The VATS can not replace open thoracotomy completely. Further study is needed,and a large multicenter randomized trial comparing VATS and thoracotomy would be ideal.

## Supporting Information

Checklist S1 **PRISMA Checklist.**
(DOC)
